# Ubiquitin control of S phase: a new role for the ubiquitin conjugating enzyme, UbcH7

**DOI:** 10.1186/1747-1028-4-17

**Published:** 2009-08-07

**Authors:** Elizabeth A Whitcomb, Allen Taylor

**Affiliations:** 1 Laboratory for Nutrition and Vision Research, JM-USDA Human Nutrition Research Center on Aging, Tufts University, 711 Washington St., Boston MA 02111, USA

## Abstract

Events within and transitions between the phases of the eukaryotic cell cycle are tightly controlled by transcriptional and post-translational processes. Prominent among them is a profound role for the ubiquitin proteasome proteolytic pathway. The timely degradation of proteins balances the increases in gene products dictated by changes in transcription. Of the dozens of ubiquitin conjugating enzymes, or E2s, functions in control of the cell cycle have been defined for only UbcH10 and Ubc3/Cdc34. Each of these E2s works primarily with one ubiquitin ligase or E3. Here we show that another E2, UbcH7 is a regulator of S phase of the cell cycle. Over-expression of UbcH7 delays entry into S phase whereas depletion of UbcH7 increases the length of S phase and decreases cell proliferation. Additionally, the level of the checkpoint kinase Chk1 increases upon UbcH7 depletion while the level of phosphorylated PTEN decreases. Taken together, these data indicate that the length of S phase is controlled in part by UbcH7 through a PTEN/Akt/Chk1 pathway. Potential mechanisms by which UbcH7 controls Chk1 levels both directly and indirectly, as well as the length of S phase are discussed and additional functions for UbcH7 are reviewed.

## Introduction

Ubiquitination of particular proteins controls many essential cellular processes by targeting the proteins for degradation [1], transport [2] or assembly into complexes [3-7]. Ubiquitin is a small 8 kDa protein that is attached to the protein substrate. An energy driven a thiol relay involving three classes of enzymes is exploited to attach ubiquitin to substrates. First, E1 proteins, of which there are very few, are charged with ubiquitin via a thiol ester linkage in an ATP-dependent process. The ubiquitin is then transferred to one of ~60 ubiquitin conjugating enzymes or E2s. Transfer of ubiquitin to the target substrate usually occurs in conjunction with an E3 ubiquitin ligase. Of the three major types of E3s, HECT (**H**omologous to **E**6-AP **C T**erminus) domain E3 ligases covalently bind the ubiquitin before passing it to the substrate. In comparison, RING (**R**eally **I**nteresting **N**ew **G**ene) domain E3s and U-box E3s, which have a modified RING domain, provide the environment for the direct transfer of ubiquitin from the participating E2 to the substrate. Several E3s are comprised of multiple subunits, some which bind substrates and others which aid in ubiquitin transfer. The combinatorial options of multiple E3s and E2s are thought to confer exquisite and extensive target specificity [1,8]. Ubiquitination can result in the attachment of a single ubiquitin, multiple mono-ubiquitins, or trees of ubiquitin which are built using one of the seven internal lysines in ubiquitin. Adding another level of diversity and biological options, the multiple lysines in ubiquitin allow the formation of many different polymer structures. The complexity of these ubiquitin polymer structures and their functions within the cell are just beginning to be elucidated [9-11].

The eukaryotic cell cycle is divided into four major phases, G1, S, G2 and M. The events within these phases and the transitions between them are tightly controlled by the timely degradation of cell cycle regulatory proteins [12-14]. Two E2 ligases have been described which are responsible for targeting for degradation a number of crucial cell cycle regulatory proteins. Each works primarily with one E3. It is likely that UbcH10 is the primary E2 that cooperates with the **A**naphase **P**romoting **C**omplex/**C**yclosome (APC/C) *in vivo *[15]. The APC/C catalyzes the ubiquitination of a number of substrates during mitosis, directing progression through mitosis and into G1 [16]. A number of substrates in G1 are also ubiquitinated via the APC/C [17,18]. Ubc3/Cdc34 is the primary E2 which works with another multi-subunit complex, consisting of the **S**kp1 and **C**ul1 proteins together with Rbx/Roc1 and one of several different **F**-box proteins (SCF) to ubiquitinate a number of cell cycle regulatory targets. The SCF is primarily responsible for controlling the G1 to S transition [19,20]. The APC/C and SCF complexes can also regulate the activity of each other. The SCF together with the β TrCP F box protein, ubiquitinates the APC/C inhibitor Emi1, and targets it for degradation [21-23], thus activating the APC/C. In addition, the APC/C complexed with the Cdh1 activator targets the F box Skp2 for ubiquitination in G1 [24,25]. The SCF^Skp2 ^complex can ubiquitinate the cyclin dependent kinase inhibitors, p27, p57 and p21 and target them for degradation, controlling the G1 to S transition. The cross regulation between these E3 ligases is but one example of the complexity of ubiquitin control of the cell cycle.

DNA replication occurs in S phase and progression through S phase is also regulated via the ubiquitin proteasome system. In order to insure that there is only one round of replication per cycle and preserve genome integrity, factors which allow replication to proceed need to be degraded after use. The DNA replication licensing factor Cdt1, binds to DNA in G1 phase at origins of replication. After replication, Cdt1 is targeted for degradation via the SCF^Skp2 ^E3 ligase complex as well as the Cul4^DDB1/Cdt2 ^E3 complex [26-28]. Another example of regulation of S phase by the ubiquitin proteasome system is the conditional turnover of the Mcm 2–7 complex, which is responsible for chain elongation and DNA unwinding [29]. However, the ubiquitin pathway components which are involved in executing the ubiquitination of this complex are not well defined [30,31].

To control the fidelity of replication, a number of proteins inhibit cell cycle progression if DNA is damaged or replication is stalled. The checkpoint kinase proteins Chk1 and Chk2, are involved in normal cell cycle progression as well as in the DNA damage repair pathway and their activity is controlled in part via the ubiquitin pathway. Thus, the activation of Chk1 via phosphorlyation by ATR after DNA damage also triggers it for ubiquitination and degradation by a CUL4 complex, assuring it is functioning for only a specified period [32,33]. The EDD E3 ligase has been shown to regulate S phase and the G2/M checkpoint through ubiquitination and degradation of the checkpoint kinase Chk2 upon DNA damage [34]. Additionally, the Fanconi Anemia complex and BRCA1/BARD1 complex, both of which are E3s, have S phase associated ubiquitination activities [35]. These complexes work together and their ubiquitination functions are essential for DNA repair [36]. Despite the identification of multiple E3s that participate in S phase events, the E2 proteins which act with these E3 ligases to control S phase progression have not been fully identified.

To enhance our understanding of the role of ubiquitin dependent proteolysis in controlling the cell cycle, we monitored the levels of different E2 proteins throughout the cell cycle [37]. Surprisingly, we found that levels of UbcH7 were regulated in a cell cycle dependent manner. A role for UbcH7 in cell cycle control *per se *had not been previously described [38] although it was reported that UbcH7 may be essential for embryonic development [39]. Specifically, we observed that UbcH7 levels declined in S phase and recovered in G2 [37,40]. Thus, we reasoned that UbcH7 might be playing a role in controlling the cell cycle. We asked whether manipulation of UbcH7 levels affected cell cycle progression. Additionally, we asked what substrates were affected by UbcH7 manipulation. We discovered that UbcH7 has a previously unappreciated role in controlling S phase of the cell cycle and discuss several possible models to explain the mechanism by which UbcH7 acts.

## Discussion

### Potential functions of UbcH7 in cell cycle control

UbcH7 has been shown to interact with a number of E3 ligases of both the HECT and RING families [41-54] with putative but as yet ill defined roles in controlling the cell cycle. UbcH7 has been shown to associate with the RING E3 Cbl [42,43]. The most well characterized Cbl target is the epidermal growth factor receptor (EGFR) signaling through which stimulates cell division. Mono-ubiquitination attenuates this signaling and directs the EGFR to the lysosome for degradation. While initial experiments suggested that UbcH7 can affect the ubiquitination of EGFR [42], controversy remains over whether UbcH7 can pass ubiquitin to Cbl targets [54]. Additionally, UbcH7 can associate with components of the SCF complex [52,53] which is well known for catalyzing the poly-ubiquitination and targeting for degradation several proteins involved in controlling the cell cycle such as the cyclin dependent kinase inhibitors p21 and p27 [19,20] and the APC/C inhibitor Emi1 [21-23]. However, it is not clear whether UbcH7 functions in conjunction with the SCF complex to affect turnover of these specific regulatory proteins. Furthermore, UbcH7 can bind to the RING E3 ligase BRCA1/BARD1 complex but does not catalyze its auto-ubiquitinating activity [54]. While BRCA1/BARD1 has a role in DNA damage repair and a likely role in an unperturbed S phase, it is unknown whether UbcH7 can affect the ubiquitination of BRCA1/BARD1 targets within cells. UbcH7 can also associate with PTEN (phosphatase and tensin homolog deleted on chromosome 10) [55] and the HECT E3, NEDD4.1, which can ubiquitinate PTEN [48]. PTEN inhibits signaling through a number of growth factor receptors, negatively controlling entry into the cell cycle. However, recently, the ability of NEDD4.1 to affect the turnover of PTEN has been called into question [56]. Thus, it is unclear whether UbcH7 can affect ubiquitination or turnover of PTEN in cells. UbcH7 can also associate with the RING E3 Parkin and may be involved in the turnover of α-synuclein [41,57] and may also affect the level of cyclin E [53]. The role of Parkin and UbcH7 in the turnover of cyclin E has not been fully characterized. UbcH7 has been co-crystallized with HECT E3 E6-AP [44,45] and UbcH7 and E6-AP can cooperate to ubiquitinate p53 in conjunction with the viral E6 protein [58]. Degradation of ubiquitinated p53 allows the progression of the cell cycle in the presence of DNA damage and the turnover of p53 by E6-AP mediated ubiquitination affects growth in oncogenic HPV infected cells. HHR23 and Mcm7, both of which have functions in S phase, have been described as substrates of E6-AP and thus may also be UbcH7 substrates [31,59]. TRIAD-1, an E3 with two RING domains, works with UbcH7 to inhibit myeloid cell growth [49]. Another dual RING domain E3 p53RFP, interacts with UbcH7 and may be involved in the turnover of the cell cycle regulatory protein p21 and play a role in apoptosis [60]. UbcH7 can associate with the HECT E3 Smurf2 and may inhibit signaling through the TGFβ receptor [50]. Signaling through TGFβ generally inhibits cell proliferation.

UbcH7 has been shown to interact with several other E3s which do not have obvious roles in the cell cycle. UbcH7, in association with the RING E3 NK-lytic associated molecule, may target urokinase like-1 protein for degradation and affect natural killer cell function [46]. UbcH7 together with the RING E3 TRAF6, ubiquitinates the neurotrophin receptor interacting factor with K63 linked chains directing its nuclear localization [51]. Thus, UbcH7 has been shown to be involved in a variety of cellular processes.

### A role for UbcH7 in S phase

**Figure 1:**
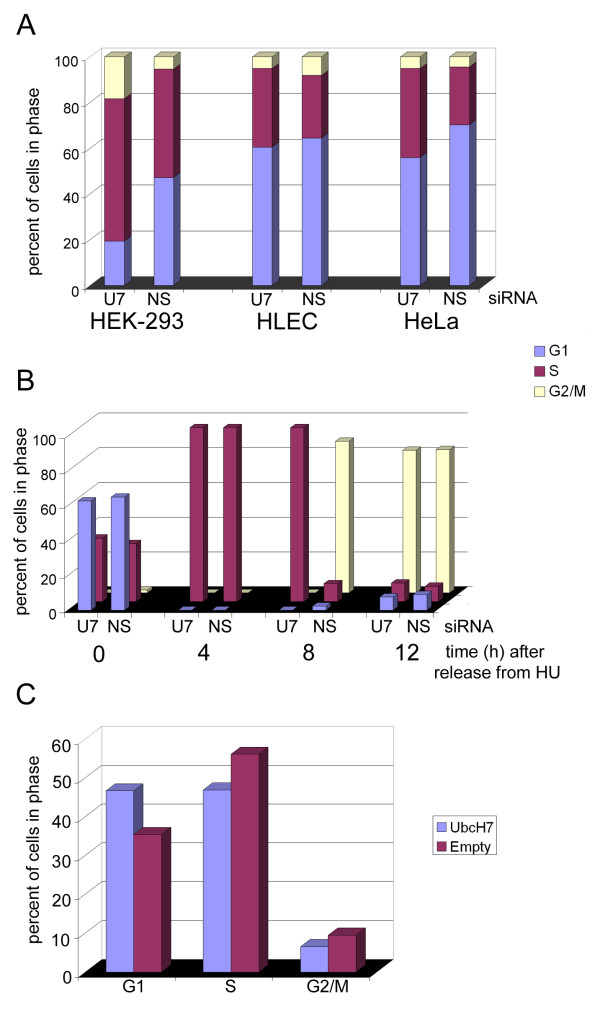
**UbcH7 levels control the entry and exit from S phase**. (A) Cells were treated with siRNA to deplete UbcH7 (U7) or a non silencing siRNA (NS) for 72 h. The cell cycle profile was determined by propidium iodide staining. (B) HeLa cells were treated for 48 h with UbcH7 specific siRNA (U7) or a non silencing siRNA (NS) as indicated. Cells were then synchronized at the G1/S boundary by treatment with 2 mM hydroxyurea for 18 h. Cells were allowed to enter cycle after removal of hydroxyurea and culture in drug-free medium. The cell cycle profile at each time point after drug removal was determined. (C) COS cells were transiently transfected with plasmids containing UbcH7 or an empty vector. After 48 h of expression, the cell cycle profile was determined as above.

We observed in both lens and HeLa cells, that UbcH7 levels declined in S phase and recovered in G2 [37,40]. Since changes in E2 levels might be expected to alter the activity of the cognate E3s, our observations of decreased UbcH7 in S phase suggested that targets of UbcH7-mediated ubiquitination may be important in regulating the progression through S phase of the cell cycle. This was confirmed upon depletion of UbcH7 using siRNA. We found an increase in the percentage of cells in S phase with three different UbcH7-siRNA sequences in multiple cell types, suggesting a common mechanism for UbcH7 action in regulating the length of S phase of the cell cycle (Fig. 1A, and see supplemental figure 1D in [40]). Further investigation using synchronized cells, confirmed that the length of S phase was increased upon UbcH7 depletion (Fig. 1B) as control cells moved from S to G2 phase during 4–8 hours after drug release while UbcH7 depleted cells were still in S phase at 8 h and didn't reach G2 until 12 h post drug release. Consistent with a slowing of S phase when UbcH7 levels are diminished, cell proliferation was decreased upon UbcH7 depletion [40]. In contrast to an increased S phase percentage upon UbcH7 depletion, over expression of UbcH7 caused an increase in the percentage of cells in G1 at the expense of S phase, suggesting a delay in entry into S (Fig. 1C) and a role for a UbcH7 target in mediating the transition from G1 into S.

### How is UbcH7 exerting its control of S phase?

**Figure 2:**
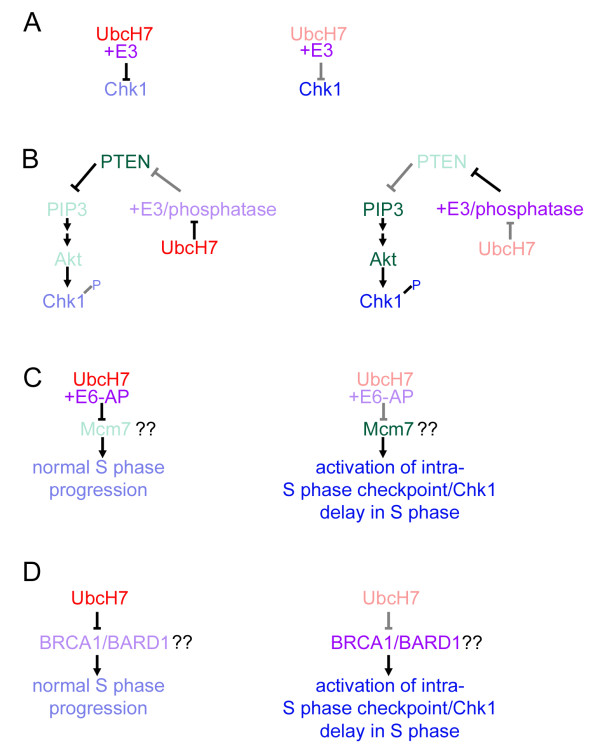
**Possible mechanisms of UbcH7 regulation of Chk1 levels sand S phase**. Conditions under normal or high levels of UbcH7 depicted on the left; after UbcH7 depletion, on the right. Decreased protein levels or decreased signaling pathways are noted by gray or lighter shading. (A) UbcH7 directly targets Chk1 for ubiquitination and degradation. If UbcH7 is directly involved in Chk1 ubiquitination, depletion of UbcH7 (right side) would result in an increase in Chk1. (B) UbcH7 increases Chk1 through a PTEN/Akt pathway. Depletion of UbcH7 (right) leads to decreased P-PTEN. Decreased activity through PTEN would increase Akt activity leading to increased P280-Chk1. The effect of UbcH7 on PTEN could be through inhibition of a phosphatase which affects the phosphorylation state of PTEN or through the inhibition of the E3 that targets PTEN for degradation. (C) UbcH7 activates Chk1 via alteration of Mcm7 levels. Decreased ubiquitination of Mcm7 through a UbcH7/E6-AP pathway (right) would lead to the imbalance of proteins in the Mcm2-7 complex. This in turn could lead to the activation of the S phase checkpoint and an increase in Chk1 levels. (D) UbcH7 depletion activates Chk1 through an increase in BRCA1/BARD1 function. The release of inhibition caused by UbcH7 (right) would lead to increased BRCA1/BARD1 ubiquitination and activation of the S phase checkpoint.

To further understand what mechanisms might be involved in the UbcH7 depletion mediated S phase delay, we examined the levels of the checkpoint kinases Chk1 and Chk2 after UbcH7 depletion. These checkpoint kinases control the intra-S phase and G2/M checkpoints upon DNA damage and also are involved in regulating progression through an unperturbed S phase. We noted that depletion of UbcH7 was associated with increased Chk1 levels, while Chk2 levels were unchanged [40]. Stabilization of Chk1 could be either a cause or a consequence of S phase delay. Possible mechanisms for UbcH7 mediated cell cycle control and Chk1 stabilization are indicated in Figure 2. Whereas under unperturbed conditions the intra-S phase checkpoint is not activated and S phase progresses normally (left side) after UbcH7 depletion (right side) Chk1 is stabilized and S phase is delayed. Pathways or substrates that are decreased are lightly shaded, while substrates that are stabilized are dark.

UbcH7 could be affecting the level of Chk1 directly via ubiquitination and targeting it to the proteasome for degradation (Fig. 2A). Chk1 is activated via phosphorylaton by ATR after genotoxic stress. This phosphorylation also targets Chk1 for degradation by the ubiquitin proteasome system [32,33] and UbcH7 can catalyze the ubiquitination of Chk1 *in vitro *(Y. Zhang, personal communication). Thus, the increase in Chk1 levels we observed upon UbcH7 depletion could be due to delayed targeting of Chk1 to the proteasome.

Alternatively, the increase in Chk1 levels could be due to stabilization through phosphorylation via Akt (Fig. 2B). Chk1 is phosphorylated at position 280 by Akt and we observed an increase of P280-Chk1 upon UbcH7 depletion [40]. Importantly, Chk1 phosphorylated at position 280 is protected from the phosphorylaton by ATR which targets it for degradation via the proteasome [61]. UbcH7 has been shown to interact with PTEN [55], a phosphatase which inhibits signaling through PI3 kinase. PI3 kinase activation leads to Akt activation. Upon UbcH7 depletion, a decrease in phosphorylated PTEN was observed [40]. Thus, decreased PTEN would lead to increased Akt activation and the increase in P280-Chk1 we observed is consistent with this. Additionally, because PTEN can delay the G1 to S transition, stabilization of PTEN levels could explain the delay in G1 to S progression observed upon UbcH7 over expression. The mechanism by which UbcH7 affects PTEN levels is unknown. Recently, NEDD4.1 was described as an E3 which targets PTEN for ubiquitination and degradation [48]. UbcH7 was one of several E2s shown to catalyze the ubiquitination of PTEN in that study. However, more recently the ability of NEDD 4.1 to affect PTEN stability has been called into question [56]. Additionally, if UbcH7 was involved in targeting PTEN for degradation, we would predict an *increase *in PTEN levels upon UbcH7 depletion. However, a decrease in PTEN levels was observed. Thus, UbcH7 may possibly be affecting the level or activity of an E3 or another factor which controls PTEN turnover. Alternatively, since UbcH7 depletion appears to decrease phosphorylated PTEN preferentially [40], UbcH7 could be affecting the level of a phosphatase that controls PTEN dephosphorylation (see Fig. 2B).

In Fig. 2C, a model for S phase extension upon UbcH7 depletion via modulation of a potential UbcH7 target is shown. *Bona fide in vivo *targets for most E3s have not been identified. It has been suggested that Mcm7 is ubiquitinated in an E6-AP dependent manner [31] and UbcH7 is one of the E2s shown to interact with E6-AP [44] and catalyze its ubiquitination function. Mcm7 is part of a complex that unwinds DNA at the replication fork and changes in Mcm7 content may affect the rate of unwinding. It is possible that UbcH7 depletion increases the level of Mcm7, turning on the intra-S phase checkpoint and thus activating Chk1. Additionally, a decrease in Mcm7, which might be predicted upon UbcH7 overexpression, would impair replication licensing and delay the progression from G1 into S phase.

In panel 2D a model for S phase extension upon UbcH7 depletion via modulation of BRCA1 activity is proposed. The BRCA1/BARD1 complex is involved in DNA damage repair and likely has functions in the progression of an unperturbed S phase. UbcH7 has been shown to bind to the BRCA1/BARD1 complex but not catalyze its auto-ubiquitination [54]. Whether the binding of UbcH7 to this complex under physiologic conditions inhibits its ability to ubiquitinate targets *in vivo *remains to be determined, but BRCA1/BARD1 activity is necessary for the intra S phase checkpoint [62]. Chk1 has been shown to be activated via BRCA1, thus if BRCA1 activity is inhibited by UbcH7, depletion of UbcH7 would increase BRCA1 activity which might in turn activate Chk1 and lead to a delay in S phase progression [63].

## Conclusion

It is clear that levels of UbcH7 are altered during the cell cycle and that alteration of UbcH7 has functional consequences with respect to controlling the length of S phase. At present, we don't know which of the models described in Figure 2 explains the changes in cell cycle progression we observe upon UbcH7 manipulation and it is important to realize that the models are not mutually exclusive. Thus, it is possible that more than one of these models is involved in orchestrating the phenomena that we've observed. Additional understanding of how UbcH7 is activated and degraded as well as identifying *bona fide *UbcH7 substrates will provide further insight into how this E2 is exerting control over cell cycle progression and should inform about how we can exploit the information to design pharmaceuticals to control cell proliferation.

## Competing interests

The authors declare that they have no competing interests.

## Authors' contributions

EW designed and carried out experiments, interpreted data and drafted the manuscript. AT participated in the design of the study, interpreted data and edited the manuscript. All authors read and approved the final manuscript.

## References

[B1] Hershko A, Ciechanover A (1998). The ubiquitin system. Annu Rev Biochem.

[B2] Carter S, Bischof O, Dejean A, Vousden KH (2007). C-terminal modifications regulate MDM2 dissociation and nuclear export of p53. Nat Cell Biol.

[B3] Kolas NK, Chapman JR, Nakada S, Ylanko J, Chahwan R, Sweeney FD, Panier S, Mendez M, Wildenhain J, Thomson TM (2007). Orchestration of the DNA-damage response by the RNF8 ubiquitin ligase. Science.

[B4] Kim H, Chen J, Yu X (2007). Ubiquitin-binding protein RAP80 mediates BRCA1-dependent DNA damage response. Science.

[B5] Wang B, Matsuoka S, Ballif BA, Zhang D, Smogorzewska A, Gygi SP, Elledge SJ (2007). Abraxas and RAP80 form a BRCA1 protein complex required for the DNA damage response. Science.

[B6] Sobhian B, Shao G, Lilli DR, Culhane AC, Moreau LA, Xia B, Livingston DM, Greenberg RA (2007). RAP80 targets BRCA1 to specific ubiquitin structures at DNA damage sites. Science.

[B7] Reddy SK, Rape M, Margansky WA, Kirschner MW (2007). Ubiquitination by the anaphase-promoting complex drives spindle checkpoint inactivation. Nature.

[B8] Pickart CM, Fushman D (2004). Polyubiquitin chains: polymeric protein signals. Curr Opin Chem Biol.

[B9] Kirkpatrick DS, Denison C, Gygi SP (2005). Weighing in on ubiquitin: the expanding role of mass-spectrometry-based proteomics. Nat Cell Biol.

[B10] Kirkpatrick DS, Hathaway NA, Hanna J, Elsasser S, Rush J, Finley D, King RW, Gygi SP (2006). Quantitative analysis of in vitro ubiquitinated cyclin B1 reveals complex chain topology. Nat Cell Biol.

[B11] Kim HT, Kim KP, Lledias F, Kisselev AF, Scaglione KM, Skowyra D, Gygi SP, Goldberg AL (2007). Certain Pairs of Ubiquitin-conjugating Enzymes (E2s) and Ubiquitin-Protein Ligases (E3s) Synthesize Nondegradable Forked Ubiquitin Chains Containing All Possible Isopeptide Linkages. J Biol Chem.

[B12] Yew PR (2001). Ubiquitin-mediated proteolysis of vertebrate G1- and S-phase regulators. J Cell Physiol.

[B13] Peters JM (2002). The anaphase-promoting complex: proteolysis in mitosis and beyond. Mol Cell.

[B14] Pines J, Lindon C (2005). Proteolysis: anytime, any place, anywhere?. Nat Cell Biol.

[B15] Jin L, Williamson A, Banerjee S, Philipp I, Rape M (2008). Mechanism of ubiquitin-chain formation by the human anaphase-promoting complex. Cell.

[B16] Bastians H, Topper LM, Gorbsky GL, Ruderman JV (1999). Cell cycle-regulated proteolysis of mitotic target proteins. Mol Biol of the Cell.

[B17] Buschhorn BA, Peters JM (2006). How APC/C orders destruction. Nat Cell Biol.

[B18] Rape M, Kirschner MW (2004). Autonomous regulation of the anaphase-promoting complex couples mitosis to S-phase entry. Nature.

[B19] Yu ZK, Gervais JL, Zhang H (1998). Human CUL-1 associates with the SKP1/SKP2 complex and regulates p21(CIP1/WAF1) and cyclin D proteins. Proc Natl Acad Sci USA.

[B20] Bornstein G, Bloom J, Sitry-Shevah D, Nakayama K, Pagano M, Hershko A (2003). Role of the SCFSkp2 ubiquitin ligase in the degradation of p21Cip1 in S phase. J Biol Chem.

[B21] Guardavaccaro D, Kudo Y, Boulaire J, Barchi M, Busino L, Donzelli M, Margottin-Goguet F, Jackson PK, Yamasaki L, Pagano M (2003). Control of Meiotic and Mitotic Progression by the F Box Protein beta-Trcp1 In Vivo. Dev Cell.

[B22] Margottin-Goguet F, Hsu JY, Loktev A, Hsieh HM, Reimann JD, Jackson PK (2003). Prophase destruction of Emi1 by the SCF(betaTrCP/Slimb) ubiquitin ligase activates the anaphase promoting complex to allow progression beyond prometaphase. Dev Cell.

[B23] Reimann JD, Gardner BE, Margottin-Goguet F, Jackson PK (2001). Emi1 regulates the anaphase-promoting complex by a different mechanism than Mad2 proteins. Genes Dev.

[B24] Wei W, Ayad NG, Wan Y, Zhang GJ, Kirschner MW, Kaelin WG (2004). Degradation of the SCF component Skp2 in cell-cycle phase G1 by the anaphase-promoting complex. Nature.

[B25] Bashir T, Dorrello NV, Amador V, Guardavaccaro D, Pagano M (2004). Control of the SCF(Skp2-Cks1) ubiquitin ligase by the APC/C(Cdh1) ubiquitin ligase. Nature.

[B26] Cang Y, Zhang J, Nicholas SA, Bastien J, Li B, Zhou P, Goff SP (2006). Deletion of DDB1 in mouse brain and lens leads to p53-dependent elimination of proliferating cells. Cell.

[B27] Zhong W, Feng H, Santiago FE, Kipreos ET (2003). CUL-4 ubiquitin ligase maintains genome stability by restraining DNA-replication licensing. Nature.

[B28] Nishitani H, Sugimoto N, Roukos V, Nakanishi Y, Saijo M, Obuse C, Tsurimoto T, Nakayama KI, Nakayama K, Fujita M (2006). Two E3 ubiquitin ligases, SCF-Skp2 and DDB1-Cul4, target human Cdt1 for proteolysis. EMBO J.

[B29] Labib K, Tercero J, eacute A, Diffley JFX (2000). Uninterrupted MCM2-7 Function Required for DNA Replication Fork Progression. Science.

[B30] Braun KA, Breeden LL (2007). Nascent Transcription of MCM2-7 Is Important for Nuclear Localization of the Minichromosome Maintenance Complex in G1. Mol Biol Cell.

[B31] Kuhne C, Banks L (1998). E3-Ubiquitin Ligase/E6-AP Links Multicopy Maintenance Protein 7 to the Ubiquitination Pathway by a Novel Motif, the L2G Box. J Biol Chem.

[B32] Zhang Y, Otterness D, Chiang G, Xie W, Liu Y, Mercurio F, Abraham R (2005). Genotoxic Stress Targets Human Chk1 for Degradation by the Ubiquitin-Proteasome Pathway. Mol Cell.

[B33] Leung-Pineda V, Huh J, Piwnica-Worms H (2009). DDB1 Targets Chk1 to the Cul4 E3 Ligase Complex in Normal Cycling Cells and in Cells Experiencing Replication Stress. Cancer Res.

[B34] Munoz MA, Saunders DN, Henderson MJ, Clancy JL, Russell AJ, Lehrbach G, Musgrove EA, Watts CK, RL S (2007). The E3 ubiquitin ligase EDD regulates S-phase and G(2)/M DNA damage checkpoints. Cell Cycle.

[B35] Taniguchi T, Garcia-Higuera I, Andreassen PR, Gregory RC, Grompe M, D'Andrea AD (2002). S-phase-specific interaction of the Fanconi anemia protein, FANCD2, with BRCA1 and RAD51. Blood.

[B36] Wang W (2007). Emergence of a DNA-damage response network consisting of Fanconi anaemia and BRCA proteins. Nat Rev Genet.

[B37] Liu Q, Shang F, Guo W, Hobbs M, Valverde P, Reddy V, Taylor A (2004). Regulation of the ubiquitin proteasome pathway in human lens epithelial cells during the cell cycle. Exp Eye Res.

[B38] Pringa E, Meier I, Muller U, Martinez-Noel G, Harbers K (2000). Disruption of the gene encoding the ubiquitin-conjugating enzyme UbcM4 has no effect on proliferation and in vitro differentiation of mouse embryonic stem cells. Biochim Biophys Acta.

[B39] Harbers K, Muller U, Grams A, Li E, Jaenisch R, Franz T (1996). Provirus integration into a gene encoding a ubiquitin-conjugating enzyme results in a placental defect and embryonic lethality. Proc Natl Acad Sci USA.

[B40] Whitcomb EA, Dudek EJ, Liu Q, Taylor A (2009). Novel Control of S Phase of the Cell Cycle by Ubiquitin-conjugating Enzyme H7. Mol Biol Cell.

[B41] Shimura H, Hattori N, Kubo S, Mizuno Y, Asakawa S, Minoshima S, Shimizu N, Iwai K, Chiba T, Tanaka K, Suzuki T (2000). Familial Parkinson disease gene product, parkin, is a ubiquitin-protein ligase. Nat Genet.

[B42] Yokouchi M, Kondo T, Houghton A, Bartkiewicz M, Horne WC, Zhan H, Yoshimura A, Baron R (1999). Ligand-induced ubiquitination of the epidermal growth factor receptor involves the interaction of the c-Cbl RING finger and ubcH7. J Biol Chem.

[B43] Zheng N, Wang P, Jeffrey PD, Pavletich NP (2000). Structure of a c-Cbl-UbcH7 complex: RING domain function in ubiquitin-protein ligases. Cell.

[B44] Huang L, Kinnucan E, Wang G, Beaudenon S, Howley PM, Huibregtse JM, Pavletich NP (1999). Structure of an E6AP-UbcH7 complex: Insights into ubiquitination by the E2-E3 enzyme cascade. Science.

[B45] Nuber U, Schwarz SE, Scheffner M (1998). The ubiquitin-protein ligase E6-associated protein (E6-AP) serves as its own substrate. Eur J Biochem.

[B46] Fortier JM, Kornbluth J (2006). NK lytic-associated molecule, involved in NK cytotoxic function, is an E3 ligase. J Immunol.

[B47] Anan T, Nagata Y, Koga H, Honda Y, Yabuki N, Miyamoto C, Kuwano A, Matsuda I, Endo F, Saya H, Nakao M (1998). Human ubiquitin-protein ligase Nedd4: expression, subcellular localization and selective interaction with ubiquitin-conjugating enzymes. Genes to Cells.

[B48] Wang X, Trotman LC, Koppie T, Alimonti A, Chen Z, Gao Z, Wang J, Erdjument-Bromage H, Tempst P, Cordon-Cardo C (2007). NEDD4-1 is a proto-oncogenic ubiquitin ligase for PTEN. Cell.

[B49] Marteijn JA, van Emst L, Erpelinck-Verschueren CA, Nikoloski G, Menke A, de Witte T, Lowenberg B, Jansen JH, Reijden BA van der (2005). The E3 ubiquitin-protein ligase Triad1 inhibits clonogenic growth of primary myeloid progenitor cells. Blood.

[B50] Ogunjimi AA, Briant DJ, Pece-Barbara N, Le Roy C, Di Guglielmo GM, Kavsak P, Rasmussen RK, Seet BT, Sicheri F, Wrana JL (2005). Regulation of Smurf2 ubiquitin ligase activity by anchoring the E2 to the HECT domain. Mol Cell.

[B51] Geetha T, Kenchappa RS, Wooten MW, Carter BD (2005). TRAF6-mediated ubiquitination regulates nuclear translocation of NRIF, the p75 receptor interactor. Embo J.

[B52] Oh KJ, Kalinina A, Wang J, Nakayama K, Nakayama KI, Bagchi S (2004). The papillomavirus E7 oncoprotein is ubiquitinated by UbcH7 and Cullin 1- and Skp2-containing E3 ligase. J Virol.

[B53] Staropoli JF, McDermott C, Martinat C, Schulman B, Demireva E, Abeliovich A (2003). Parkin is a component of an SCF-like ubiquitin ligase complex and protects postmitotic neurons from kainate excitotoxicity. Neuron.

[B54] Brzovic PS, Keeffe JR, Nishikawa H, Miyamoto K, Fox D, Fukuda M, Ohta T, Klevit R (2003). Binding and recognition in the assembly of an active BRCA1/BARD1 ubiquitin-ligase complex. Proc Natl Acad Sci USA.

[B55] Waite KA, Eng C (2003). BMP2 exposure results in decreased PTEN protein degradation and increased PTEN levels. Hum Mol Genet.

[B56] Fouladkou F, Landry T, Kawabe H, Neeb A, Lu C, Brose N, Stambolic V, Rotin D (2008). The ubiquitin ligase Nedd4-1 is dispensable for the regulation of PTEN stability and localization. Proceedings of the National Academy of Sciences.

[B57] Shimura H, Schlossmacher MG, Hattori N, Frosch MP, Trockenbacher A, Schneider R, Mizuno Y, Kosik KS, Selkoe DJ (2001). Ubiquitination of a new form of alpha-synuclein by parkin from human brain: implications for Parkinson's disease. Science.

[B58] Ciechanover A, Shkedy D, Oren M, Bercovich B (1994). Degradation of the tumor suppressor protein p53 by the ubiquitin-mediated proteolytic system requires a novel species of ubiquitin-carrier protein, E2. J Biol Chem.

[B59] Kumar S, Talis AL, Howley PM (1999). Identification of HHR23A as a substrate for E6-associated protein-mediated ubiquitination. Journal of Biological Chemistry.

[B60] Huang J, Xu LG, Liu T, Zhai Z, Shu HB (2006). The p53-inducible E3 ubiquitin ligase p53RFP induces p53-dependent apoptosis. FEBS Lett.

[B61] Puc J, Keniry M, Li HS, Pandita TK, Choudhury AD, Memeo L, Mansukhani M, Murty VV, Gaciong Z, Meek SE (2005). Lack of PTEN sequesters CHK1 and initiates genetic instability. Cancer Cell.

[B62] Xu B, Kim S-t, Kastan MB (2001). Involvement of Brca1 in S-Phase and G2-Phase Checkpoints after Ionizing Irradiation. Mol Cell Biol.

[B63] Lee EY (2002). BRCA1 and Chk1 in G2/M Checkpoint: A New Order of Regulation. Cell Cycle.

